# DNLC: differential network local consistency analysis

**DOI:** 10.1186/s12859-019-3046-4

**Published:** 2019-12-24

**Authors:** Jianwei Lu, Yao Lu, Yusheng Ding, Qingyang Xiao, Linqing Liu, Qingpo Cai, Yunchuan Kong, Yun Bai, Tianwei Yu

**Affiliations:** 1 School of Software Engineering, Tongji University, Shanghai, China; 2 Institute of Advanced Translational Medicine, Tongji University, Shanghai, China; 3 Department of Environmental Health, Emory University, Atlanta, GA USA; 4 Department of Biostatistics and Bioinformatics, Emory University, Atlanta, GA USA; 5 Department of Pharmaceutical Sciences, School of Pharmacy, Philadelphia College of Osteopathic Medicine, Georgia Campus, Suwanee, GA USA

**Keywords:** Biological network, Gene expression, Local Moran’s I

## Abstract

**Background:**

The biological network is highly dynamic. Functional relations between genes can be activated or deactivated depending on the biological conditions. On the genome-scale network, subnetworks that gain or lose local expression consistency may shed light on the regulatory mechanisms related to the changing biological conditions, such as disease status or tissue developmental stages.

**Results:**

In this study, we develop a new method to select genes and modules on the existing biological network, in which local expression consistency changes significantly between clinical conditions. The method is called DNLC: Differential Network Local Consistency. In simulations, our algorithm detected artificially created local consistency changes effectively. We applied the method on two publicly available datasets, and the method detected novel genes and network modules that were biologically plausible.

**Conclusions:**

The new method is effective in finding modules in which the gene expression consistency change between clinical conditions. It is a useful tool that complements traditional differential expression analyses to make discoveries from gene expression data. The R package is available at https://cran.r-project.org/web/packages/DNLC.

## Background

The biological system operates by tightly controlling the abundance and activity of thousands of proteins. The regulations and interactions can be summarized as a scale-free network [1–3]. The known networks summarized from existing knowledge, e.g. protein-protein interaction and signal transduction networks, are static in nature. Yet in real biological systems, the activities of the edges on the network are dynamic [4]. In the context of gene expression, nodes on the biological network correspond to genes. The expression levels of genes that are close on the network can change between states of correlated, uncorrelated, or even reversely correlated, depending on the biological condition [5]. Currently, a number of methods can analyze gene expression data in the context of an existing biological network. Most of the methods try to find “network markers”, i.e. small subnetworks that change expression levels in response to clinical conditions [6–17]. Some other methods study the dynamic correlation patterns on the network, without considering the clinical outcome [18–20].

Given the biological network is dynamic, and physiological conditions influence the activity of the edges in the network, it is natural to consider the change in expression consistency, i.e. the co-expression patterns in subnetworks, in response to changing physiological states. So far, no method is available to find changes of expression consistency on the network. In this manuscript, our goal is to develop a computational method to detect genes around which the expression consistency changes significantly in response to physiological states. Finding such genes can reveal important mechanisms related to disease development, by revealing biological functions that become more tightly regulated or de-regulated in association with disease status. Such a method should be able to complement existing differential expression methods to shed new light on the data.

For this purpose, we borrow the measure of Local Moran’s I (LMI) from the field of spatial statistics, which quantifies spatial auto-correlation on a map [21]. We treat the network as a map, and calculate LMI for each node based on its expression value and the expression values of nearby nodes on the network. We then use the LMI values to quantify the local expression consistency around any given node. A high positive LMI value of a node in a specific sample implies that the node has a similar expression value to its neighbors in that sample, and their expression values are either very high or very low. In contrast, a large negative LMI value means the node is a spatial outlier, i.e. a node that has low consistency with its surrounding nodes on the network [22]. By combining LMI scores with the clinical data, and using regression models with local false discovery rate correction [23], our method finds nodes around which local expression consistency change significantly between different clinical conditions. It showed promising result in both simulations and real data analyses.

## Methods

### Calculating local Moran’s I (LMI) score on the network

**Fig. 1:**
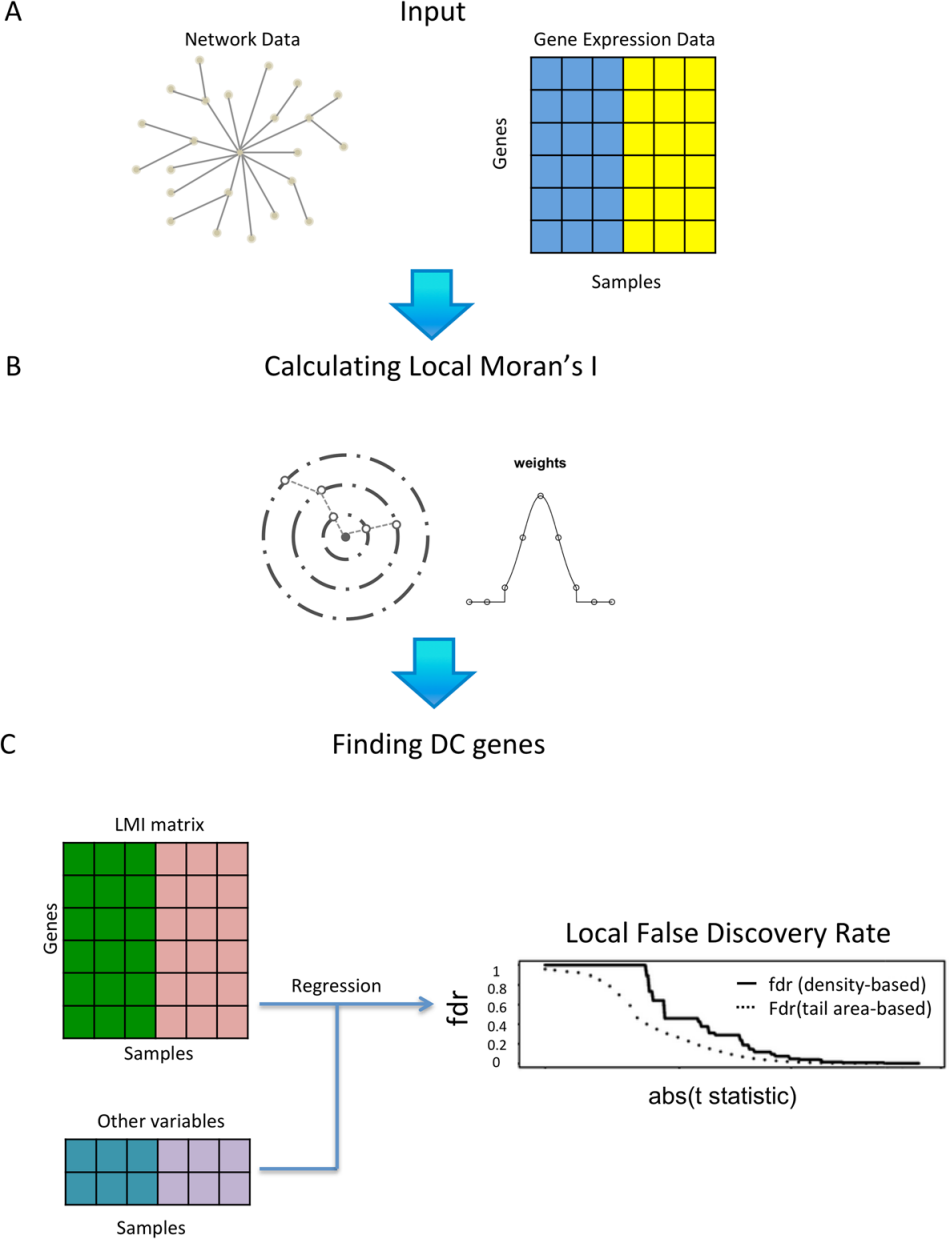
The overall workflow of our method. **a** The input data structure; **b** Calculating LMI scores for each gene; **c** Finding DC genes

The overall workflow of the method is shown in Fig. 1. The data contains four pieces: ***M***_***p × N***_ is the gene expression matrix with *p* genes in the rows and *N* samples in the columns; ***y*** is the clinical outcome vector of length *N*; ***G = (V, E)*** is the network between the *p* genes, where the vertices ***V*** correspond to the genes, and the edges ***E*** represent functional relations between the genes; ***C***_***m × N***_ is the matrix of other clinical variables, such as age, gender *etc*, with *m* variables in the rows and *N* samples in the columns. We assume there is a one-to-one match between the genes in the matrix and the nodes in the network. Any unmatched genes/nodes are eliminated from the analysis. To prepare for the analysis, the expression matrix is normalized using normal score transformation for every gene.

We calculate the LMI score for every gene in each sample. The goal of LMI is to quantify the extent to which nodes that are close to a given node have expression values similar to it. The formula of LMI for gene *i* in sample *k* is:

$$ {I}_{i,k}=\frac{z_{i,k}-\overline{z_k}}{\sigma_k^2}{\sum}_{j\ne i}{w}_{ij}\left({z}_{j,k}-\overline{z_k}\right), $$

where *z*_*i*, *k*_ is the expression of gene *i* in sample *k*, $\overline{z_k}$ is the average gene expression in sample *k*, *z*_*j*, *k*_ is the expression of gene *j* for all the other genes on the network (where *j* ≠ *i*); ${\upsigma}_k^2$ is the variance of expression in sample *k*; *w*_*ij*_ is the weight assigned to gene *j*, which depends on its distance to gene *i* on the network.

There can be many strategies for the calculation of weights. The goal is to focus on the small region surrounding gene *i* on the network. One strategy is to assign the inverse of the distance *d*_*ij*_ between gene *i* and gene *j* as *w*_*ij*_. Another strategy is to determine *w*_*ij*_ using a distance threshold: genes within a distance are given the same weight, while those farther away are given the weight of 0. In this study, we use a truncated Gaussian function to assign the weights,

$$ {w}_{ij}=\left\{\begin{array}{c}\frac{1}{\sqrt{2\pi }}{e}^{-{d}_{ij}^2/2},{d}_{ij}\le 2\\ {}0,{d}_{ij}>2\end{array}\right., $$

Where *d*_*ij*_ is the length of the shortest path between nodes *i* and *j*. The weights are then normalized such that for gene *i*, ∑_*j* ≠ *i*_*w*_*ij*_ = 1.

The intuition of the approach is as follows: for a given node *i*, only nodes in its vicinity receive substantial weights. Then the calculation of *I*_*i*, *k*_ essentially takes a weighted sum of the products of $\left({z}_{i,k}-\overline{z_k}\right)$ and all the nodes in the vicinity $\left({z}_{j,k}-\overline{z_k}\right)$, normalized by the variance of the expression levels in the sample. We can see that when $\left({z}_{i,k}-\overline{z_k}\right)$ and most of the $\left({z}_{j,k}-\overline{z_k}\right)$ are of the same sign, and have large absolute values, *I*_*i*, *k*_ will have a large positive value. On the other hand, when $\left({z}_{i,k}-\overline{z_k}\right)$ and most of the $\left({z}_{j,k}-\overline{z_k}\right)$ are of opposite sign, and have large absolute values, then *I*_*i*, *k*_ will be negative with a large absolute value. When there is no expression consistency between the nodes near node *i*, or if their values are close to zero, *I*_*i*, *k*_ will be close to zero. Thus the LMI value *I*_*i*, *k*_ is a good measure of the expression consistency of node *i* with its network vicinity.

### Selecting differential consistency (DC) genes

After computing *I*_*i*, *k*_ for every node *i* in every sample *k*, we have a matrix with the LMI values. The dimension of this LMI matrix is exactly the same as the original gene expression matrix, with *p* genes in the rows and *N* samples in the columns. We then find if a gene’s LMI score changes significantly between different clinical conditions, while incorporating confounders such as age, race etc.

The procedure here is similar to traditional differential expression analysis where confounders are considered (Table 1). The relationship between the clinical outcome, the LMI score of a gene, and confounders can be described by a generalized linear model:

$$ \mathrm{E}\left(\mathrm{y}\ |\mathrm{LMI}\_\mathrm{Score},\mathrm{Confounders}\right)={g}^{-1}\left(\upalpha \times \mathrm{LMI}\_\mathrm{Score}+{\sum}_m{\upbeta}_m\times {\mathrm{Confounder}}_m\right), $$

where *g*^*−1*^*(·)* is an inverse link function, which can be chosen according to the specific type of the outcome variable. In this study we use the logistic regression for binary outcome variable, and Cox proportional hazards model for survival outcome variable.

**Table 1:** The pseudocode for conducting DC gene search on the network

Input: ***G*** (gene network), ***M*** (expression matrix), ***y*** (clinical outcome vector), *T* (local fdr threshold), ***C*** (confounder matrix) Output: Collection of DC genes: S Standardize each row of ***M*** Local Moran’s I Matrix ***I*** = Local _ Moran _ I(***G***, ***M***) For each node *i* in ***G*** do *t*_*i*_ = t statistic from generalized linear model ***y***~***I***_***i***_ + ***C*** End for Fit {*t*_*i*_}_*i* = 1, …, *p*_ to mixture model using local fdr to find {*lfdr*_*i*_}_*i* = 1, …, *p*_ *S* = {*i* : *lfdr*_*i*_ ≤ *T*} Return *S*	

After the t-statistics for the parameter α for all genes are calculated, we follow the local false discovery rate (lfdr) procedure to adjust for multiple testing. For most genes, their local consistency on the network are unrelated to the clinical outcome, and their t-statistics will approximately follow a normal distribution. Genes around which local expression consistency change significantly between clinical conditions will have more extreme t-statistic values. Thus, we can consider the t-statistics of all the genes to follow a mixture model with two components:

$$ f(t)={\pi}_0{f}_0(t)+\left(1-{\pi}_0\right){f}_1(t), $$

where *f* is the mixture density for the observed *t*-statistics of all the genes, *f*_0_ and *f*_1_ are the densities of the *t*-statistics of the null (non-DC) and non-null (DC) genes respectively, and π_0_ is the proportion of null genes [23]. We can estimate the probability that each gene belongs to the non-null category using mixture density estimation. In this study, we use the R package locfdr for the calculation [24]. By setting a threshold for the lfdr value, we can distinguish DC genes from the others.

### Finding network communities of DC genes

After selecting the DC genes, we use a simple and efficient algorithm to group the DC genes and their directly connected genes into network communities for better data interpretation. We adopt the fast-greedy algorithm that directly optimizes modularity score to get the communities of a large graph [25]. After detecting several communities among the DC genes and their neighbors, biological function analysis is performed on each detected community. We use the GOstats method [26], which is based on the Gene Ontology biological processes, to perform the analysis.

## Results

### Simulation study

We conducted a systematic study using simulated data. In each simulation, data was generated using the following steps.

1. A scale-free network with *m* nodes was generated using the Barabasi-Albert model [27]. Based on this network structure, we calculated the ***Σ*** matrix, in which *Σ*_*i,j*_*=* ${c}^{d_{i,j}}$, where *c* was a constant between 0 and 1, and d_*i*, *j*_ was the shortest path between nodes *i* and *j* on the network.
2. An *m* × *n* gene expression matrix was generated using the multivariate normal distribution, using ***Σ*** as the variance-covariance matrix.
3. We then randomly selected five nodes from the network, the degree of which were within a certain range. Among the nodes within two hops of these five nodes, we changed the elements of the ***Σ*** matrix to *Σ*_*i,j*_*=* ${b}^{d_{i,j}}$, where *b* was a constant between 0 and 1, and d_*i*, *j*_ was the shortest path between nodes *i* and *j*.
4. Another *m* × *n* gene expression data matrix was generated using the multivariate normal density, using the modified ***Σ*** matrix as the variance-covariance matrix.
5. We joined the two matrixes horizontally to obtain the simulated expression matrix, which was of dimension *m* × 2*n*, where *m* was the number of genes and 2*n* was the total number of samples.
6. The outcome variable corresponding to first *n* samples (original correlation samples) were set to 0, and the last *n* samples (changed correlation samples) were set to 1.

Four parameters were used to control the signal strength of the data: (a) The base correlation *c*, which controlled the background correlation strength. Four levels were used (base correlation = 0.2, 0.4, 0.6, 0.8). (b) The changed correlation *b*. We applied four levels (changed correlation = 0.2, 0.4, 0.6, 0.8) for simulation. (c) The degrees of the five selected nodes. Two ranges (degrees between 5 to 10, and between 15 to 20) were used. (d) Sample size in the simulated expression data (number of samples = 50, 100, 200, 300, 400, 500, 700, 1000).

Fifty datasets were simulated at each parameter setting. After a dataset was generated, we used three methods to analyze the data: (1) the DNLC algorithm; (2) the differential network analysis (DNA) method [28], and (3) simple differential expression (DE) analysis using t-test and local fdr correction. We evaluated the results by the PR-AUC (area under the precision-recall curve). Each node was assigned a status depending on the generation process: 1 (changed correlation with neighbors) or 0 (unchanged correlation with neighbors). The local fdr values calculated by each method were used as the predictor variable to generate the precision-recall curve. In each setting, the average area under the curve (AUC) was calculated to reflect the efficacy that the nodes with true local expression consistency changes were differentiated from other nodes.

**Fig. 2:**
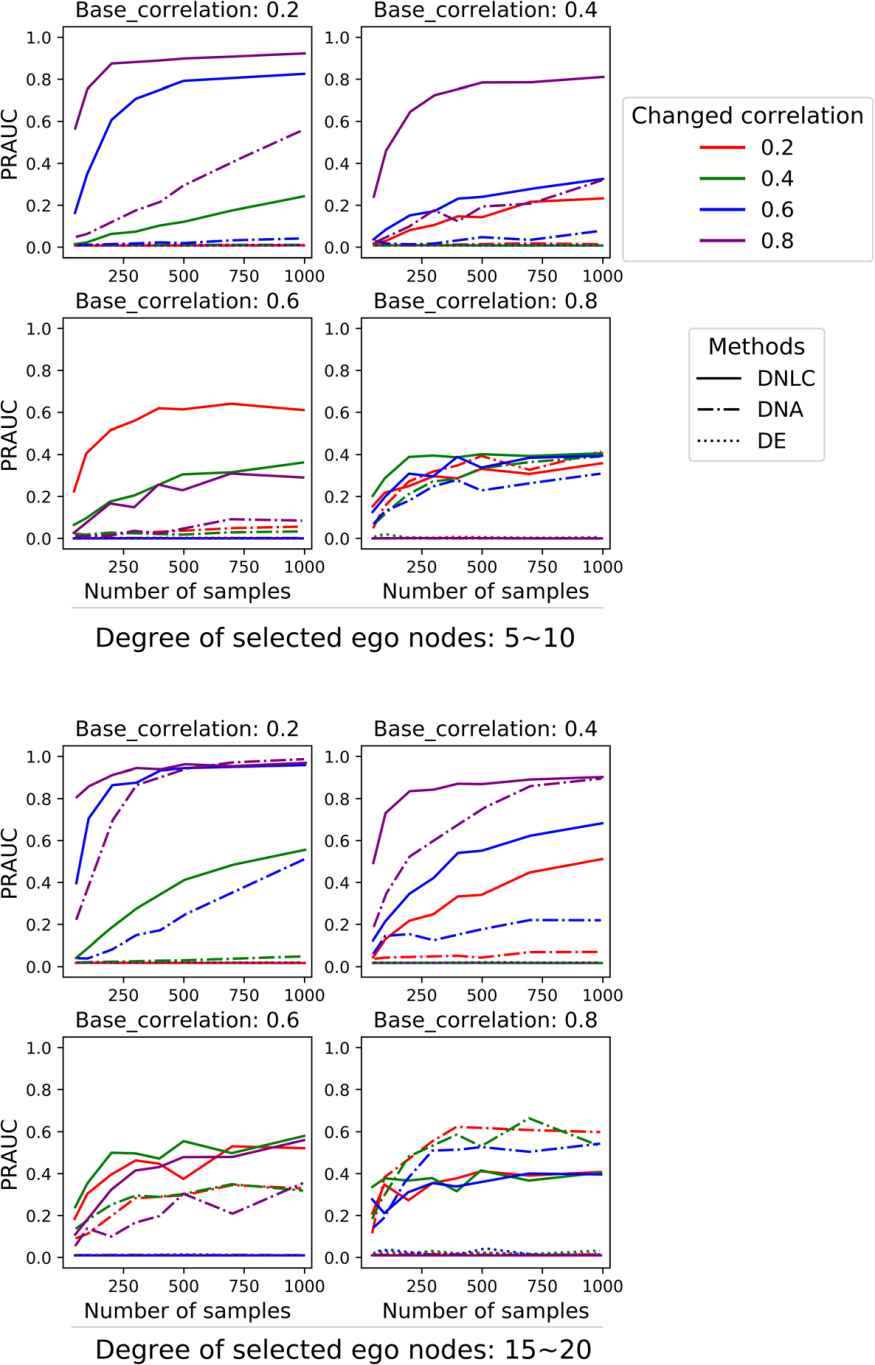
Simulation results. The PR-AUC are plotted against the sample sizes. Each data point represents the average result of 50 simulations

As shown in Fig. 2, when the base correlation level *c* was equal to the changed correlation level *b*, at all settings the PR-AUC values were close to zero (Fig. 2). On the other hand, when the base correlation level *c* was different from the changed correlation level *b*, the PR-AUC values increased with the sample size, both in the cases of *b* > *c* and in the cases of *b* < *c*. When the base correlation *c* was high (0.6 or 0.8), the power to detect the DC nodes was limited (Fig. 2, second and fourth columns). On the other hand, when the base correlation was at low or medium level (0.2 or 0.4), which was close to real data situations, the power was reasonably high when the difference between *b* and *c* was high (Fig. 2, first and third columns). In comparison, testing for differential expression didn’t detect the differential consistency on the network in most cases. The differential network analysis (DNA) method exhibited some power to detect network differential local consistency, but its AUC values were lower than the DNLC method in most cases, except when the base correlation was high, and the network density was high (Fig. 2, lower-right panel). Although the DNA method seeks differential correlation, it doesn’t use network information, which was likely the reason of the inferior performance. Overall, the simulation results validated that the DNLC method was effective in separating the nodes around which the expression consistency were changed.

### Real data analysis: GSE10255

To test our method, we used the High-quality Interaction (HINT) database [29] for the human biological network. Two publicly available expression datasets were analyzed. The first dataset was the gene expression dataset of acute lymphoblastic leukemia (ALL) subjects in response to methotrexate (MTX) treatment (GSE10255) [30]. The dataset contained 12,704 rows (genes) and 161 columns (samples). The clinical outcome variable was the reduction of circulating leukemia cells after MTX treatment. At the lfdr threshold of 0.2, a total of 510 DC genes were selected. Furthermore, network modules were detected among the selected genes and their immediate neighbors on the network. In the following discussion, we focus on the largest module. The plots and functional annotations of all the modules are available at web1.sph.emory.edu/users/tyu8/DNLC/MTX.

**Fig. 3:**
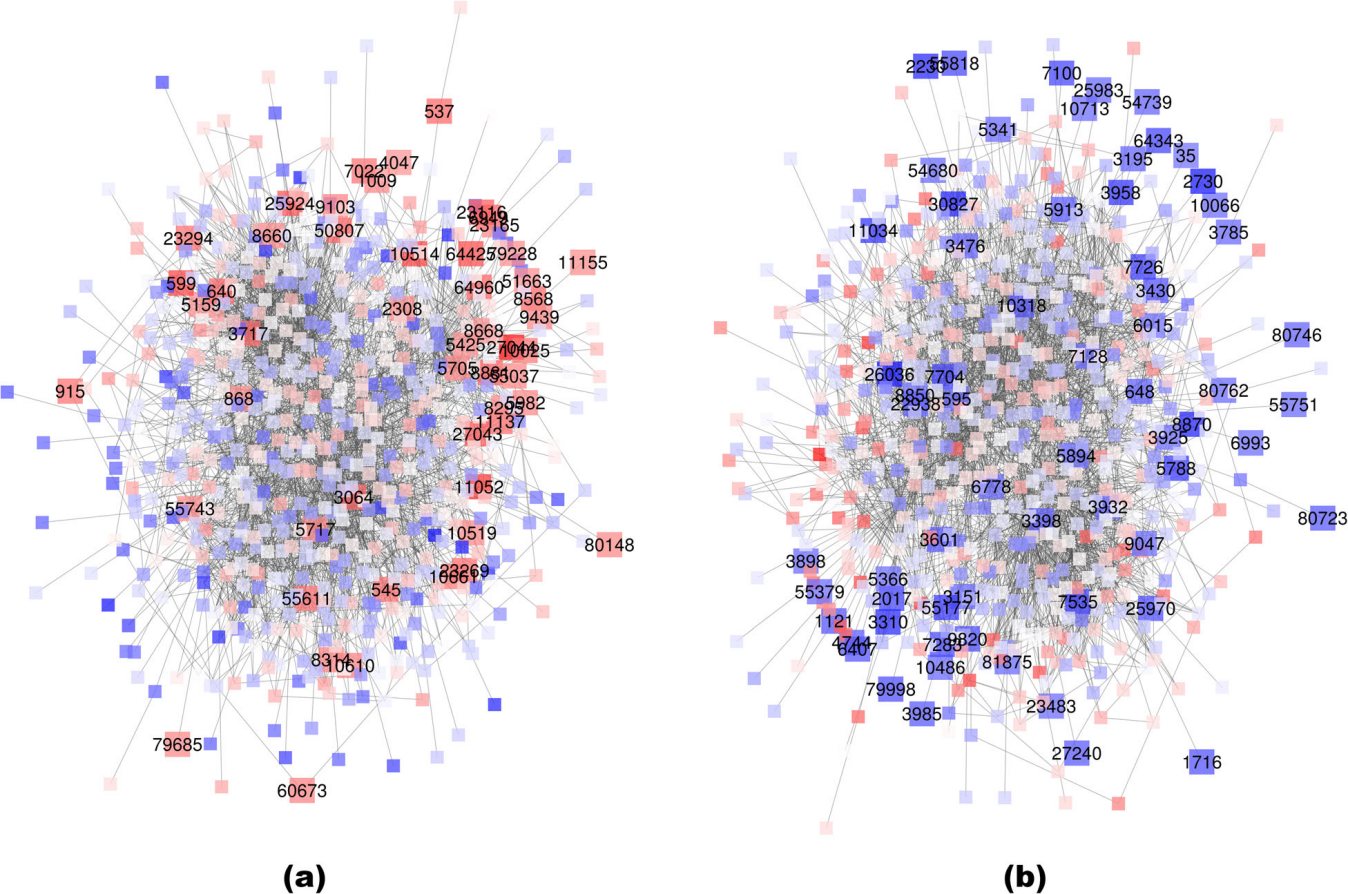
The first module from the GSE10255 dataset. **a** genes with LMI positively associated with MTX response (red); **b** genes with LMI negatively associated with MTX response (blue). Entrez gene IDs are used in the plots

We used the GOStats package to find gene ontology terms that were over-represented by the lists of genes [26]. For the largest network module (Fig. 3a), the biological processes overrepresented by the positive DC genes, i.e. genes with increased local consistency in patients with higher MTX response, could be categorized into five major groups: phosphorylation and ubiquitination; peptide hormone secretion; catabolic process; DNA synthetic and repairing; apoptosis and response to hyperoxia. All these functions are closely related to MTX sensitivity in ALL. It has been well-documented that genes that regulate protein modification, apoptosis and DNA synthesis/repair influence caner development [31]. Both phosphorylation and ubiquitination of proteins have been reported to play important roles in MTX resistance in leukemia treatment. Phosphorylation of HSC70, an MTX-binding protein, regulates the transportation of MTX into the cells and contributes to MTX resistance in L1210 leukemia cells [32]. It has also been demonstrated that the MTX chemotherapeutic effect can be significantly reduced by antiepileptic drugs due to the downregulation of reduced folate carrier (Rfc1) activity, regulated by the ubiquitin-proteasome pathway [33]. Among the selected genes by our method, genes 868 (CBLB), 5705 (PSMC5) and 5717 (PSMD11) regulate protein modifications. Many research demonstrated the role of CBLB in leukemia [34, 35], while PSMC5 and PSMD11 were only reported to be involved in cancer development in very recent studies [36–38].

We next focus on the genes that regulate hormone secretion [39], catabolic process [40], and hyperoxia [41], whose roles in ALL treatment are not self-evident. The genes that regulate peptide/protein secretion interfere with the efficacy of chemotherapy to cancer cells by regulating hormone levels. It has been reported that the secretion of peptide hormones are changed in ALL [42]. In addition, some papers reported that insulin and insulin-like factor (IGF) secretions affect the resistance of chemotherapy in ALL patients [43]. Among the selected genes, genes 640 (BLK proto-oncogene), 3717 (JAK2), 8660 (IRS2) and 25,924 (MYRIP) are major genes involved in peptide secretions. JAK2 mutation is believed to have prognostic impact in acute myeloid leukemia [44–46]. The BLK proto-oncogene is involved in leukemia development [47]. IRS2 is an adaptor protein associated with the receptor of erythropoietin, insulin-like growth factor 1. Defective IRS2 expression plays a role in impaired hematopoietic cell differentiation [48] .

The selected DC genes also included genes that regulate protein catabolic process. It has been reported that resistance to methotrexate (MTX) in leukemia is related to hydrolase and thymidylate synthase activities, which are catabolic processes [49]. Among the selected genes, 2308 (FOXO1) and 5707 (PSMD1) are regulators of the catabolic process. It has been reported that FOXO1 can play a role in the development of acute myeloid leukemia (AML) [50]. Currently, there are no report about the relationship between PSMD1 and leukemia. However, PSMD1 has been reported to be overexpressed in other cancers [51].

The negative DC genes, i.e. genes with decreased local consistency in patients with higher MTX response (Fig. 3b), were also clearly related to cancer development and progressions. The over-represented GO terms by the negative DC genes include immune cell development and activation [52, 53]; apoptosis [54]; mammary gland epithelium cell proliferation [55, 56]; cell-cell adhesion [57], and cell depolymerization [58]. A number of the selected DC genes are known to affect ALL progression. Also, some of them are known to regulate MTX resistance in leukemia treatment. For example, our method selected genes 595 (CCND1) and 3398 (ID2) that regulate mammary gland epithelial cell proliferation. It has been reported that CCND1 G870A polymorphism is associated with the risk of leukemia and toxicity of MTX in ALL [59, 60]. ID2 is known to be associated with chemotherapy response and prognosis in acute myeloid leukemia [61].

### Real data analysis: TCGA BRCA dataset

We applied the method to a second data set, the breast cancer (BRCA) gene expression dataset from The Cancer Genome Atlas (TCGA). We used the Cox proportional hazards model to link gene LMI values with patient survival outcome, while adjusting for baseline demographic variables including age, gender, and ethnicity. The plots and functional annotations of all the modules are at web1.sph.emory.edu/users/tyu8/DNLC/BRCA. Again we focus on the largest modules for the discussion here.

**Fig. 4:**
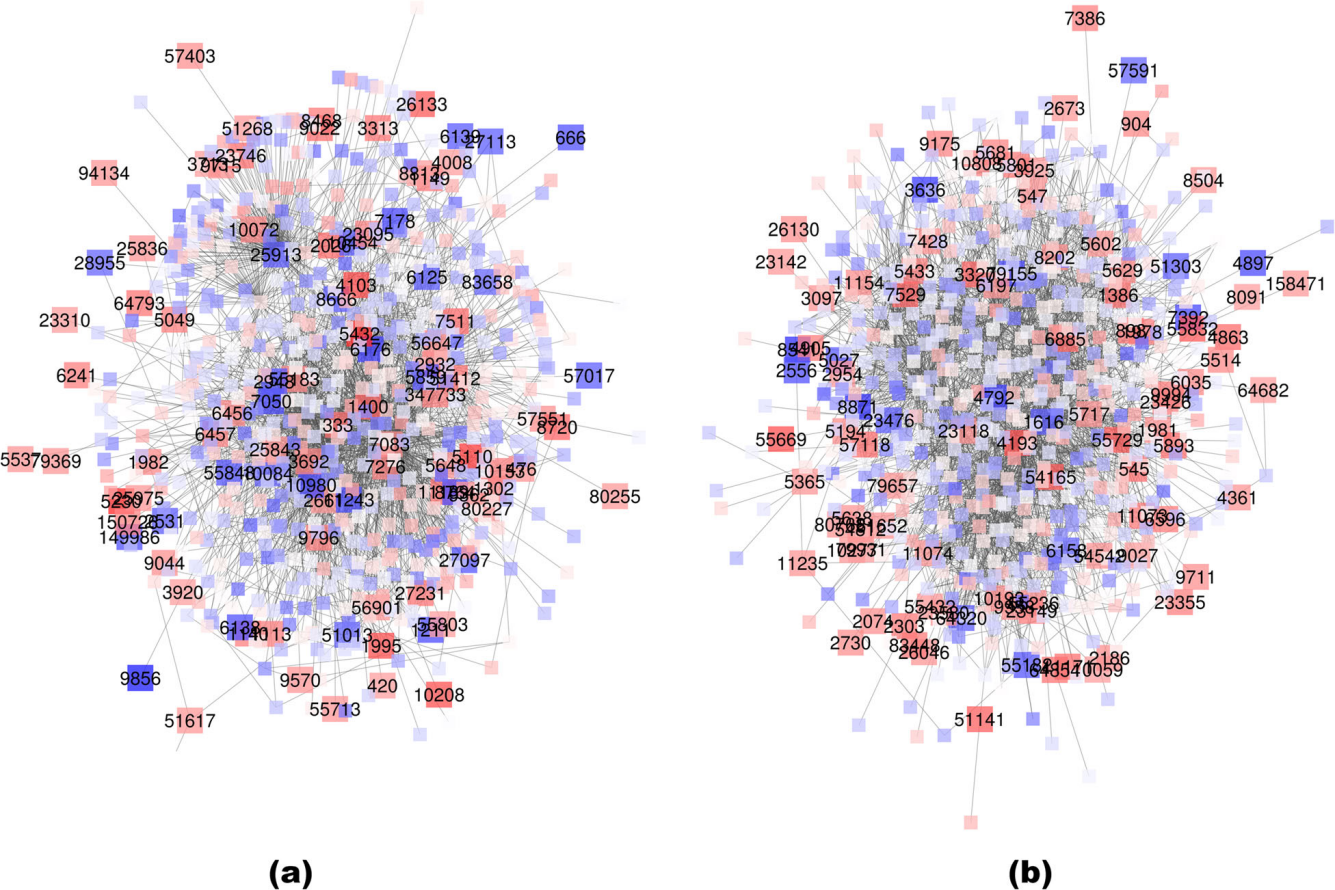
The first two modules from TCGA BRCA data. **a** module 1; **b** module 2. Red: LMI positively associated with survival; blue: LMI negatively associated with survival. Entrez gene IDs are used in the plots

In the first module (Fig. 4a), the negative DC genes, i.e. genes with decreased local consistency in patients with lower risk, appear to be more functionally coherent. The biological processes over-represented by the negative DC genes include protein/peptide metabolic process, biogenesis, or membrane targeting and transport, which are obviously related to breast cancer development. As examples, genes 6125 (RPL5) and 6138 (RPL15) were among the most significant genes in the list. RPL5 has been reported to be a tumor suppressor gene in breast cancer development [62]. While there is no research paper reporting the role of RPL15 in breast cancer, one study suggested the methylation of RPL15 may be involved in cancer development [63]. Genes 333 (APLP1), 476 (ATP1A1), 1113 (CHGA), and 2026 (ENO2) were on the positive gene list. ATP1A1 has been previously reported to be over-expressed in breast cancer [64]. The overexpression CHGA, a neuroendocrine carcinomas marker, characterizes 10% of infiltrative breast cancer [65]. ENO2 is used as a biomarker to help identify neuroendocrine differentiation in breast cancer [66].

In module 2 (Fig. 4b), the majority of the positive genes were involved in protein ubiquitination, which is a critical process in cancer development [67]. Functional groups of the negative genes include I-Kappa B kinase signaling. Nuclear factor kappa-beta (NF-kappaB) is a transcription factor that modulates the expression of many genes involved in cell proliferation, differentiation, apoptosis and metastasis. Nuclear factor-kappa B is used as a predictor of treatment response in breast cancer [68]. Expression of nuclear factor kappa B (NF-κB) is also used as a predictor of pathologic response to chemotherapy in patients with locally advanced breast cancer [69]. In the I-Kappa B kinase signaling pathway, our method found genes 4792 (NFKBIA), 23,476 (BRD4), and 79,155 (TNIP2) to be significantly associated with breast cancer survival. One study investigated common variants within the gene coding region for NF-kappaB and IkappaB, NFKB1 and NFKBIA, for involvement in sporadic breast cancer. However, the results did not support an involvement of the NFKBIA polymorphisms in sporadic breast cancer in the Caucasian population [70].

The local consistencies of genes 3636 (INPPL1) and 5027 (P2RX7) were also found to be negatively associated to breast cancer survival. They regulate phospholipid dephosphorylation and transport. INPPL1 is also known as SHIP2, which is involved in breast cancer development [71–73]. P2RX7 is also known as P2X7. Purinergic signaling has been implicated in the regulation of many cellular processes and is involved in tumor development and metastasis. Reports revealed that the activation of the P2X7 receptor promotes breast cancer cell invasion and migration, and the P2X7 receptor may be a useful therapeutic target for the treatment of breast cancer [74].

## Discussions

In this manuscript, we presented a new method to detect differential consistency (DC) genes on the biological network, as well as network modules where DC genes are concentrated. By using the Local Moran’s I (LMI) for measuring local expression consistency on the network, and using the regression framework, the method is versatile enough to be able to study continuous, categorical, and survival outcomes.

**Fig. 5:**
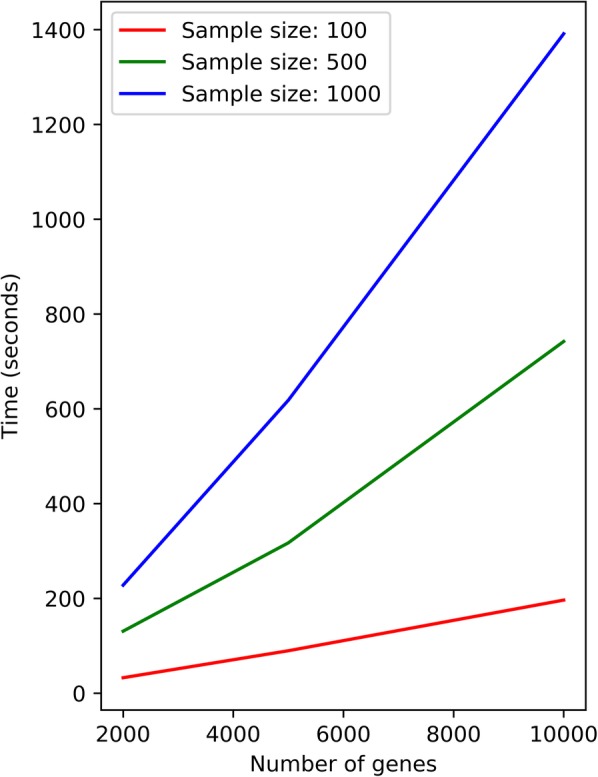
The computing time of the DNLC method. The computing time was recorded on a Lenovo Think Station P9000 with Xeon E5–2630 CPU, using a single thread for computing

Given a large-scale network containing thousands of nodes, the number of possible sub-networks is astronomical. Thus we take the approach of focusing on a specific type of subnetwork: the ego-network, which is defined by the neighborhood of a given node [11]. This approach reduces the number of sub-networks to the number of nodes in the network. The algorithm focuses on the relations between the center node of each subnetwork to its surrounding nodes, using the LMI to measure their expression consistency. The computing time of the method increases roughly linearly with the number of genes (nodes) and the sample size (Fig. 5). For example, with 10,000 genes and 500 samples, the method costs roug hly 12 min using single thread computation.

## Conclusion

In simulations and real data analyses, we have shown the method is effective in finding genes around which expression consistency changes in response to the clinical outcome. The method is a useful tool that complements traditional differential expression type of analyses to make discoveries from gene expression data.

## Data Availability

The R package is available at https://cran.r-project.org/web/packages/DNLC.

## Abbreviations

ALL: Acute lymphoblastic leukemia
BRCA: Breast invasive carcinoma cohort
DC: Differential Consistency
HINT: High-quality Interaction database
lfdr: local false discovery rate
LMI: Local Moran’s I
PR-AUC: Area under the precision-recall curve
TCGA: The Cancer Genome Atlas
